# Emergence of functional neuromuscular junctions in an engineered, multicellular spinal cord-muscle bioactuator

**DOI:** 10.1063/1.5121440

**Published:** 2020-04-28

**Authors:** C. D. Kaufman, S. C. Liu, C. Cvetkovic, C. A. Lee, G. Naseri Kouzehgarani, R. Gillette, R. Bashir, M. U. Gillette

**Affiliations:** 1 Neuroscience Program, University of Illinois at Urbana-Champaign, Urbana, Illinois 61801, USA; 2 Beckman Institute for Advanced Science and Technology, University of Illinois at Urbana-Champaign, Urbana, Illinois 61801, USA; 3 Holonyak Micro and Nanotechnology Laboratory, University of Illinois at Urbana-Champaign, Urbana, Illinois 61801, USA; 4 College of Medicine, University of Illinois at Urbana-Champaign, Urbana, Illinois 61801, USA; 5 Department of Bioengineering, University of Illinois at Urbana-Champaign, Urbana, Illinois 61801, USA; 6 Department of Molecular and Integrative Physiology, University of Illinois at Urbana-Champaign, Urbana, Illinois 61801, USA; 7 Department of Cell and Developmental Biology, University of Illinois at Urbana-Champaign, Urbana, Illinois 61801, USA

## Abstract

Three-dimensional (3D) biomimetic systems hold great promise for the study of biological systems *in vitro* as well as for the development and testing of pharmaceuticals. Here, we test the hypothesis that an intact segment of lumbar rat spinal cord will form functional neuromuscular junctions (NMJs) with engineered, 3D muscle tissue, mimicking the partial development of the peripheral nervous system (PNS). Muscle tissues are grown on a 3D-printed polyethylene glycol (PEG) skeleton where deflection of the backbone due to muscle contraction causes the displacement of the pillar-like “feet.” We show that spinal cord explants extend a robust and complex arbor of motor neurons and glia *in vitro*. We then engineered a “spinobot” by innervating the muscle tissue with an intact segment of lumbar spinal cord that houses the hindlimb locomotor central pattern generator (CPG). Within 7 days of the spinal cord being introduced to the muscle tissue, functional neuromuscular junctions (NMJs) are formed, resulting in the development of an early PNS *in vitro*. The newly innervated muscles exhibit spontaneous contractions as measured by the displacement of pillars on the PEG skeleton. Upon chemical excitation, the spinal cord-muscle system initiated muscular twitches with a consistent frequency pattern. These sequences of contraction/relaxation suggest the action of a spinal CPG. Chemical inhibition with a blocker of neuronal glutamate receptors effectively blocked contractions. Overall, these data demonstrate that a rat spinal cord is capable of forming functional neuromuscular junctions *ex vivo* with an engineered muscle tissue at an ontogenetically similar timescale.

## INTRODUCTION

Biological robotics is a growing field that derives inspiration from biological systems for real world applications. Challenges that have historically plagued more traditional, rigid robotics include interacting with biological tissue, self-repair, and collapsing into biodegradable parts after completion of a task.^1^ Biology has already solved many of these problems faced by rigid robots in creative ways. By abstracting and recapitulating these solutions, we will be able to replicate increasingly natural, complex motor behaviors with novel engineering approaches to biorobotics.^2^

Mimicking how organisms actuate is one approach that has already led to bio-inspired devices and machines.^3–7^ Recent work on biological soft robots has already produced “biobots” that recapitulate a variety of locomotive behaviors, e.g*.,* crawling, swimming, walking, and jumping.^4,8–15^ These locomotive biohybrid actuators are produced primarily with either cardiac or skeletal muscle and may also use flexible materials such as aluminum, shape metal alloys, hydrogels,^12,14^ and soft plastics.^2,3,16–18^ Cardiac muscle provides rhythmic contractions without requiring external input, but the intrinsic frequency of those cells is not easily modified, thereby limiting the scope of potential behaviors. Skeletal muscle allows for a wider array of potential behaviors but requires extrinsic control mechanisms, such as electric fields, optogenetics, or chemical stimulation.^7,14,19–23^

Previous work on skeletal muscle has commonly used C2C12 myoblasts to study muscle differentiation, force production, and neuromuscular interactions *in vitro*.^24–28^ C2C12 is also the most common cell line used when developing biohybrid machines.^9,13,29–32^ Research has demonstrated that engineered C2C12-derived muscle tissues maintain a consistent degree of contraction for over 250 days after seeding.^19^ Actuation independent of the experimenter influences requires a wireless, onboard control unit. Vertebrates have solved this through neural control of muscle tissues. Some previous work has bypassed motor neuron input to the muscle entirely through extrinsic control mechanisms, such as applied electric fields,^5^ optogenetics,^14,23,31^ or chemical stimulation.^5,19,23^ However, neurons and muscles exhibit different phenotypes and behaviors when in monoculture or when cultured together.^20^ Therefore, to create an *in vitro* model of the neuromuscular junction (NMJ), it is important to co-culture these cells to allow for emergent organization and multicellular interactions to occur *in vitro*.

In vertebrates, complex locomotor tasks are primarily controlled by spinal cord and brainstem networks. While these networks are heavily modulated by the brainstem, most of the pattern and rhythm generation involved with locomotion are housed within the spinal cord. An intact rat spinal cord has three distinct anatomical regions from rostral to caudal: cervical, thoracic, and lumbar.^33^ The lumbar enlargement is a widened area of the caudal spinal cord that serves as the attachment site for nerves of the lower limbs. Central pattern generators (CPGs) are complex, oscillatory networks within the spinal cord, which govern a range of rhythmic actions from locomotion to breathing.^20^ Quadrupedal locomotion requires the coordination of flexor-extensor muscle pairings simultaneously within a limb and between ipsi- and contralateral pairs of limbs. This is performed by spinal circuits known as CPGs. CPGs are roughly symmetrical circuits that generally consist of lateral excitatory interneurons, medial inhibitory interneurons, and outputs to ventrolateral cholinergic motor neurons. The function of a CPG is to produce patterned output from non-patterned input. Previous work has identified the first and second lumbar vertebrae (L1–L2) as the location of the hind-limb locomotor CPG in rats.^33^

Embryonic stem cell (ESC)-derived neuron-like cells can interact with skeletal muscle to cause small contractions.^19,23^ However, stem cell-derived neurons share only a subset of known characteristics with rodent motor neurons and contain cell types in varying ratios that are not fully identified.^34,35^ Human induced pluripotent stem cells (hIPSCs) have been used to form three-dimensional (3D) NMJs that contain many of the sub-cellular constituents of *in situ* NMJs.^30,36,37^ While the activity of stochastically formed neuronal networks can demonstrate synchronous activity,^38^ functional *in situ* neuronal circuits are highly organized and serve specific purposes. The processes of natural embryonic development, which shape the spinal cord, are more robust than current stem cell differentiation protocols, and the resulting circuits are more consistent and well-characterized. The rat spinal cord contains approximately 36 × 10^6^ cells, of which over 10 × 10^6^ are neurons.^39^ It is beyond current capabilities to reproduce such a complex, multicellular system using embryoid bodies (EBs), organoids, or other stem cell-derived neural tissues.

Here, we use a mixture of top-down and bottom-up design principles to take advantage of the intrinsic locomotor circuitry of the spinal cord and generate patterned contractions of a self-assembled, 3D muscle tissue by chemical stimulation of an isolated, intact locomotor CPG. Bottom-up design of the muscle allows us to develop a tissue that has an appropriate size to interface with a rat spinal cord while also minimizing necrosis.^13^ Utilizing top-down design principles, we interface an intact locomotor CPG to drive muscle contraction with the engineered muscle tissue to produce a multi-cellular system capable of undergoing spinally driven muscle contraction. We first developed a method to culture a rat spinal cord explant *in vitro* such that it extends a robust arbor of motor neurons and further optimized it for co-culture with C2C12-derived myoblasts. We then confirmed the presence of pre- and post-synaptic structural components of a motor unit on the 3D striated muscle. Finally, we showed that while the muscle contracts spontaneously, the contractile frequency is controllable through the application and subsequent blockade of the neurotransmitter applied to the spinal cord. Neurochemical stimulation of the spinal cord generated patterned contractions of the muscle, suggesting the functionality of the CPG. This spinobot is a novel biohybrid robot with multicellular architecture that demonstrates spinal cord-driven muscle contractions.

## RESULTS

**FIG. 1.**
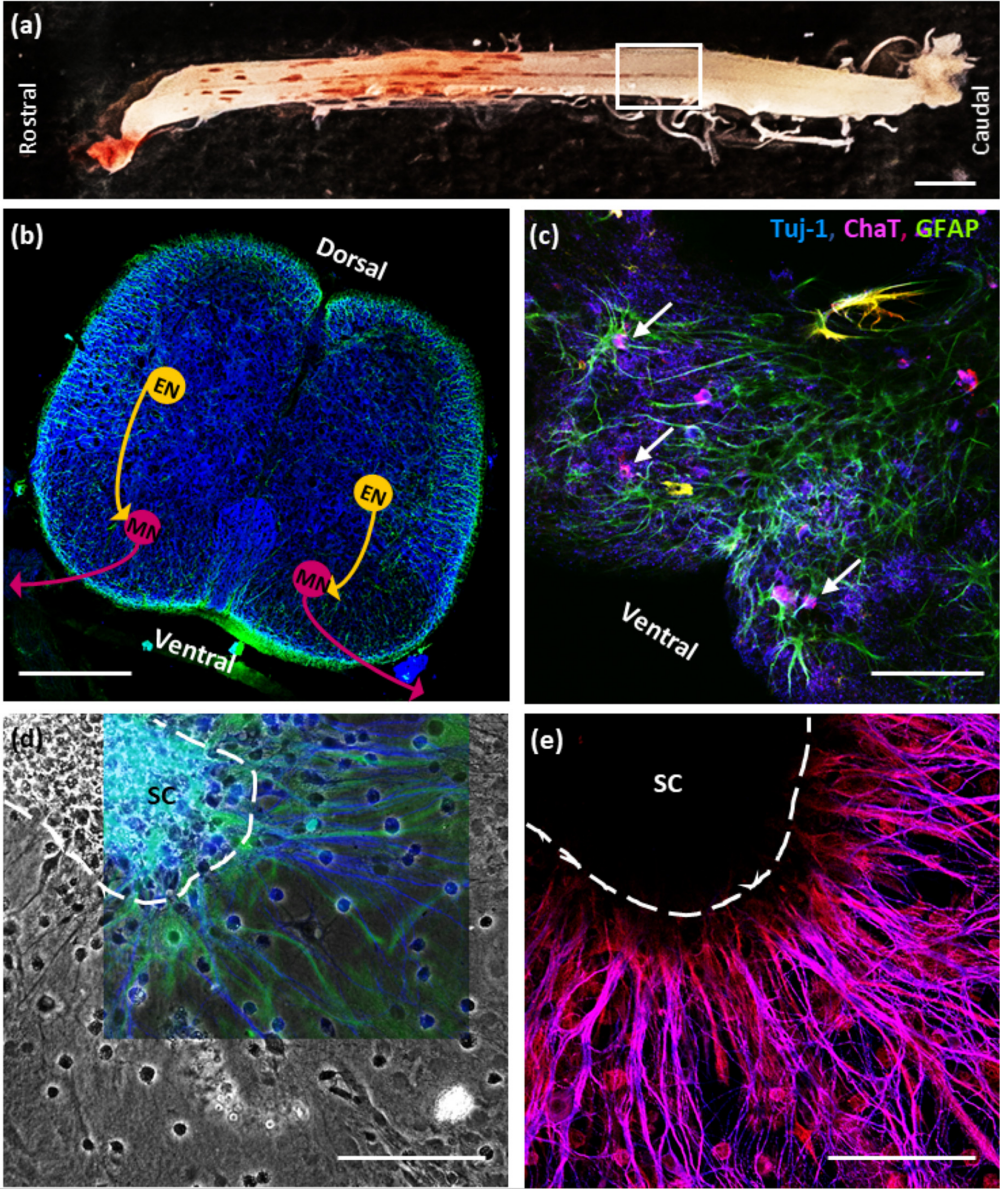
Organotypic culture of an intact neonatal rat spinal cord. (a) A fully intact rat spinal cord is shown, with a white box outlining the first and second lumbar (L1–L2) regions, which houses the hindlimb locomotor central pattern generator (CPG). (b) Horizontal cross section of a lumbar spinal cord shows the presence of neurons (Tuj-1, blue) and glia (GFAP, green) within the spinal cord. Schematic of excitatory interneurons (ENs) stimulating lower motor neurons (MNs) in the ventrolateral spinal cord. (c) Choline acetyltransferase (ChAT, magenta) co-localizes with Tuj-1 and appears ventrolaterally in the cross section, confirming the presence of the cholinergic neurons. (d) Neuronal outgrowth radiates from a 7 DIV rat spinal cord (SC) cultured on Matrigel-coated glass. This outgrowth contained large populations of both neurons (Tuj-1, blue) and astrocytes (GFAP, green). (e) The colocalization of ChAT and Tuj-1 tubulin immunohistochemistry indicates that these processes are nearly entirely cholinergic. The dark area is SC that is out of the plane of focus.

Neonatal rat spinal cords extend a robust arbor of glia and cholinergic neurons *in vitro*. To develop a biobot with an onboard neural system capable of performing complex locomotor tasks, we isolated and cultured a segment of neonatal (P1–5) spinal cord (SC) from within the first and second lumbar (L1–L2) vertebrae. This region was selected because an intact SC is approximately 23.5 mm in length [Fig. 1(a)], over fourfold longer than the biobot skeleton. Previous work had shown that this was the location of the hindlimb locomotor CPG (Cazalets *et al.*, 1995).^33^ Figure 1(b) shows a simplified diagram of a locomotor central pattern generator with an inhibitory oscillatory center that regulates the firing of excitatory interneurons and their downstream motor neurons. In vertebrates, these motor neurons in the ventral horn serve as CPG outputs that innervate and control muscle contraction [Fig. 1(b)].

We surgically isolated the L1–L2 region and cultured it on Matrigel-coated glass for up to 22 days *in vitro* (DIV). In all cases, the spinal cord was cultured on the ventral side down with the goal of inducing the motor neurons of the ventral horn [Figs. 1(b) and 1(c)] to extend out of the spinal cord. When cultured on Matrigel, the spinal cord extended robust process outgrowth [Fig. 1(d)]. This complex arbor of extensions was composed of many cell types, including not only neurons but also glia [Fig. 1(d)], which are important for the formation and maintenance of functional synapses.^40^ Of the neuronal processes that are extended by 7 DIV, a large majority expressed choline acetyltransferase (ChAT), an enzyme found exclusively in ACh-producing neurons [Fig. 1(e)]. Additionally, electrophysiological recordings reveal that cultured spinal cords produce electrical activity both spontaneously and when stimulated with glutamate (GLUT) (Fig. S1). Thus, spinal cords extend a robust arbor of electrically active, cholinergic neurons that are likely to be motor neurons due to their location within the spinal cord, as well as the robust presence of ChAT. This indicated that a spinal cord explant could serve as a viable system for muscular interaction and control.

**FIG. 2.**
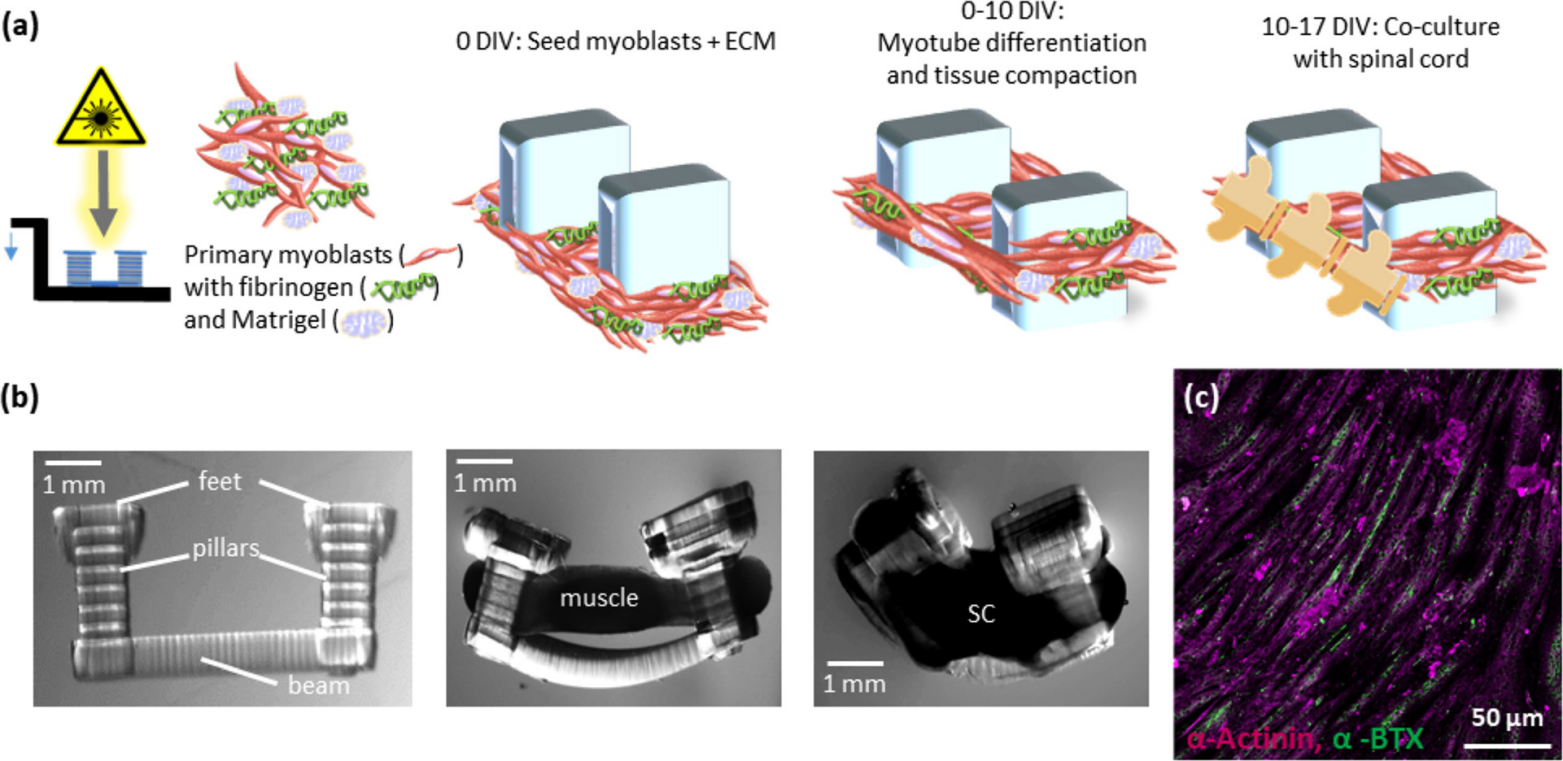
Methodology for building a spinobot from the spinal cord and C2C12 myoblasts. (a) Top: a stereolithography apparatus (SLA) is used to polymerize hydrogel structures in an additive process. A cell-ECM mixture of C2C12 myoblasts, thrombin, fibrinogen, and Matrigel is seeded onto the 3D-printed skeleton. Over 10 DIV, myotubes compact the ECM and begin to differentiate. At 10 DIV, a segment of lumbar spinal cord is seeded onto the spinal cord and co-cultured for an additional 7 days. (b) Lateral step-by-step view of the multicellular spinobot construction. Left*:* a hydrogel skeleton is 3D printed using a stereolithography apparatus (SLA). Middle: muscle attached to the hydrogel skeleton causes bending by generating passive tension. Right: co-culture of spinal cord (SC) and muscle strip over the beam of the hydrogel skeleton. (c) 2D culture of C2C12 myoblasts forms aligned myotubes over 10 days on untreated glass. Alpha-actinin marks the skeletal muscle (magenta), and nicotinic AChR clusters (alpha-bungarotoxin, green) can be visualized along the whole length.

Fabrication of a spinobot skeleton and seeding of C2C12 and spinal cord components to form a spinobot. First, we 3D-printed a poly (ethylene glycol) diacrylate (PEGDA) hydrogel skeleton composed of two pillars connected by a flexible beam [Figs. 2(a) and 2(b)]. The pillars serve as attachment points much like tendons within the musculoskeletal system. A gel composed of myoblasts and extracellular matrix (ECM) proteins (Matrigel, thrombin, and fibrinogen) is seeded around the pillars to form a solid muscle strip [Figs. 2(a) and 2(b)]. As the myocell-ECM gel solidifies and the muscle cells differentiate, they cause the pillars to be pulled closer together due to passive tension [Fig. 2(b), middle panel]. An L1–L2 spinal cord segment was placed along the longitudinal axis of the muscle strip when the muscle reached 10 DIV, and they were cultured for an additional 7 DIV [Fig. 2(b), right panel]. When cultured on glass, C2C12-derived myotubes roughly align with each other and express acetylcholine receptors (AChRs) distributed along their full length [Fig. 2(c)].

**FIG. 3.**
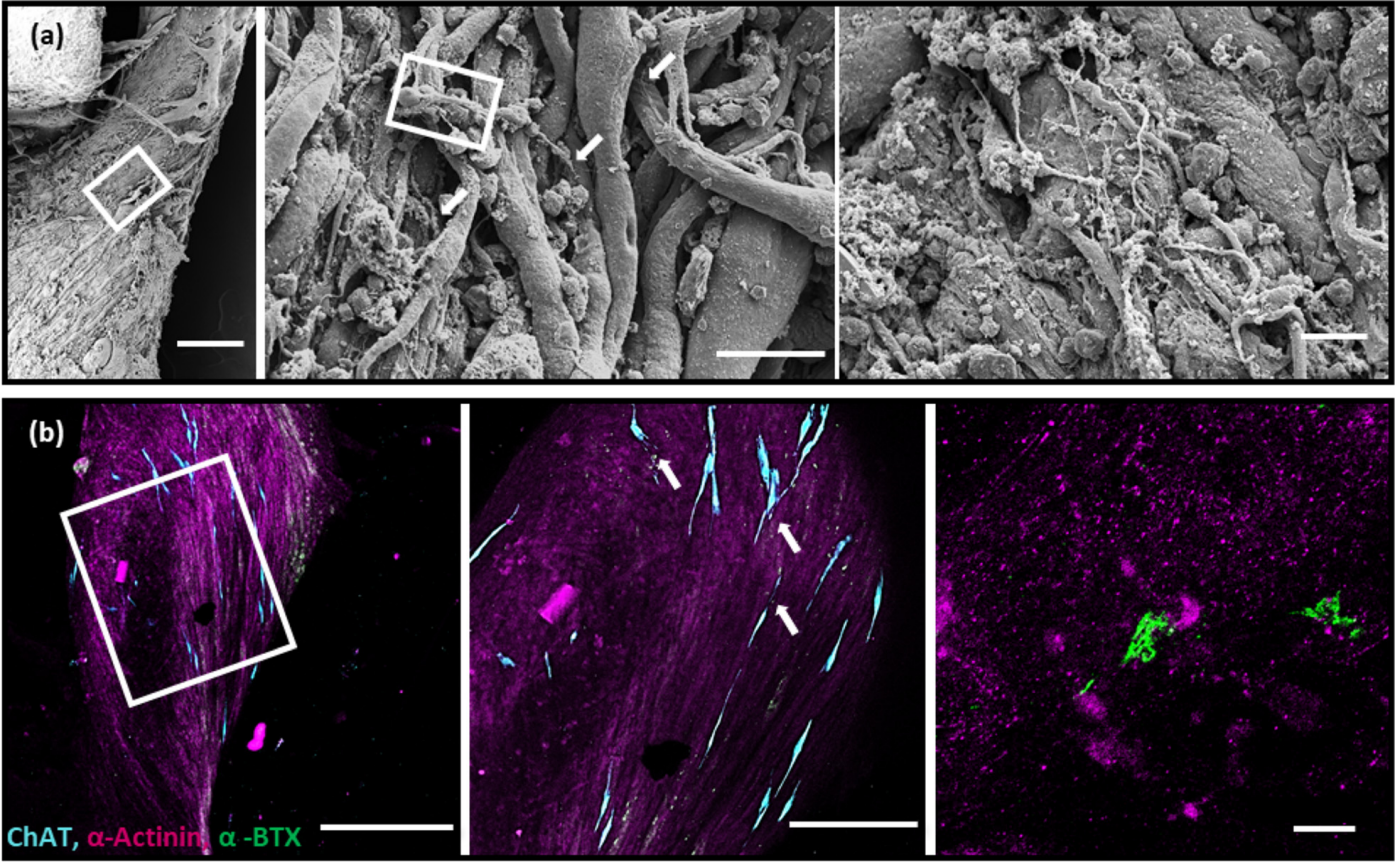
Structural evidence of abutting cholinergic neurons and postsynaptic acetylcholine receptors. (a) SEM images of the inferior side of the spinobot at increasing magnification from left to right. Scale bars are 200 *μ*m, 25 *μ*m, and 20 *μ*m, respectively. Myotubes appear aligned, and neuronal processes lie in close apposition to muscle fibers. Neuronal processes extend beyond the surface layer of myotubes into deeper muscle tissue, which are indicated by the arrows. (b) Photomicrographs of the immunohistochemically stained spinobot at increasing magnification from left to right: Scale bars are 500 *μ*m, 250 *μ*m, and 10 *μ*m, respectively. Left: muscle fibers (α-actinin, magenta) align along the longitudinal axis of the spinobot. AChR clusters (α-bungarotoxin, green) are present, but largely clustered around the cholinergic neurons (ChAT, cyan). Middle: acetylcholine receptors and ChAT-positive neurons are found to be localized close together, as indicated by arrows. Right: the development of typical pretzel-like motor end plate structures on the postsynaptic muscle indicates the formation of structural neuromuscular junctions.

3D co-culture of the spinal cord and engineered muscle tissue form structural motor units. High resolution, high magnification scanning electron microscopy (SEM) images of the spinobot revealed that neurites extended from the spinal cord into the muscle tissue [Fig. 3(a), left panel]. The neurites not only were observed at the surface of the tissue but were also found to be aligned with deeper muscle fibers [Fig. 3(a), middle and right panels]. AChRs were found to be clustered at specific locations along the myotubes, and these locations overlapped with the presence of cholinergic neurons [Fig. 3(b), middle panel]. In areas where no ChAT-positive cells were found, there was a notable lack of receptors [Fig. 3(b), left panel]. This suggests the possibility of bi-directional neuron-muscle communication *in vitro* that mimics what occurs *in vivo*.^41^ Furthermore, the structure of the AChR clusters revealed the characteristic pretzel-like shape of an NMJ [Fig. 3(b), right panel].^42^ The degree of complexity noted at this motor end plate matches the previous literature for a motor end plate approximately 7 days after the first contact between neurons and muscle *in vivo*. The close apposition of neurons and muscle fibers, the co-localization of cholinergic neurons with their receptors on the muscle, and the presence of pretzel-shaped AChR clusters are consistent with the presence of neuromuscular junctions within the 3D multicellular system.

**FIG. 4.**
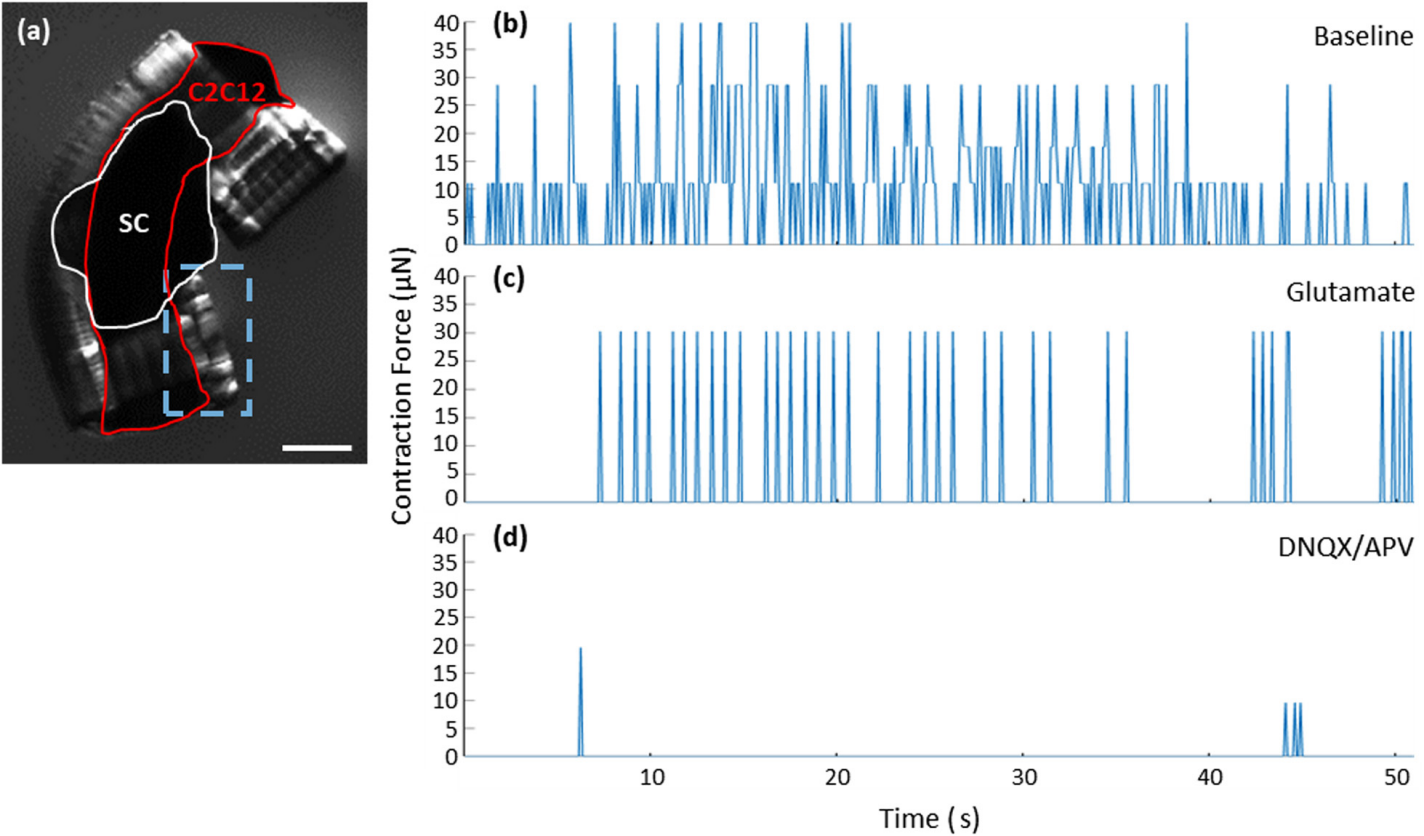
Spontaneous contractions of the spinobot can be controlled by neurochemical modulation. (a) Lateral image of a spinobot. The red outline shows the muscle, the white outline indicates the spinal cord placement, and the dashed blue box indicates the region of interest for video capture of pillar displacement. The scale bar is 1 mm. (b)–(d) Representative traces of contraction force under baseline (b), stimulation (c), and inhibition (d) conditions. As shown in (b), the muscle contracts spontaneously with variable frequency and magnitude at the baseline. When glutamate is added to the media (c), contractions become more consistent in strength and frequency. (d) After washing out the glutamate, the addition of glutamate receptor antagonists (DNQX and APV) together with more glutamate results in a nearly complete reduction in both the number and the frequency of contractions.

Muscle contractions are driven by spinal cord stimulation and can be blocked by inhibiting excitatory neuronal firing. The functionality of the spinal cord-muscle system was tested under baseline (BL), stimulated, and inhibited conditions [Fig. 4(a), Movies S2–S4] and recorded by video. Initially, the spinobot was placed in co-culture medium (CCM) and provided no additional modulatory cues during the baseline recording, where we observed spontaneous muscle contractions that generated 10–40 *μ*N of active tension across the beam [Fig. 4(b)]. The addition of 300 *μ*M glutamate to the solution caused a distinct change in the pattern of muscle contraction, with glutamate-induced contractions occurring with more consistent magnitudes and in a more patterned manner [Fig. 4(c)]. Spinal cord-driven contractions exhibited a maximum force of ∼41 *μ*N, with the mean contractile force of 61% (25 *μ*N). When less than 200 *μ*M glutamate was added, there was no clear change in the pattern of electrical activity produced by cultured spinal cords [Figs. S1(b) and S1(c)]. The addition of glutamate-receptor antagonists (20 *μ*M DNQX to block AMPA receptors and 50 *μ*M APV to block NMDA receptors)^43^ resulted in a near complete cessation of muscle contraction, even with additional glutamate application [Fig. 4(d)]. The observation that the twitching did not completely cease under glutamate inhibition is expected as skeletal muscles have some degree of spontaneous contraction both *in vivo* and *in vitro*.^44,45^ The DNQX/APV application caused inhibition beyond baseline levels, indicating that the spinal cord was driving the majority of the observed spontaneous contractions.

**FIG. 5.**
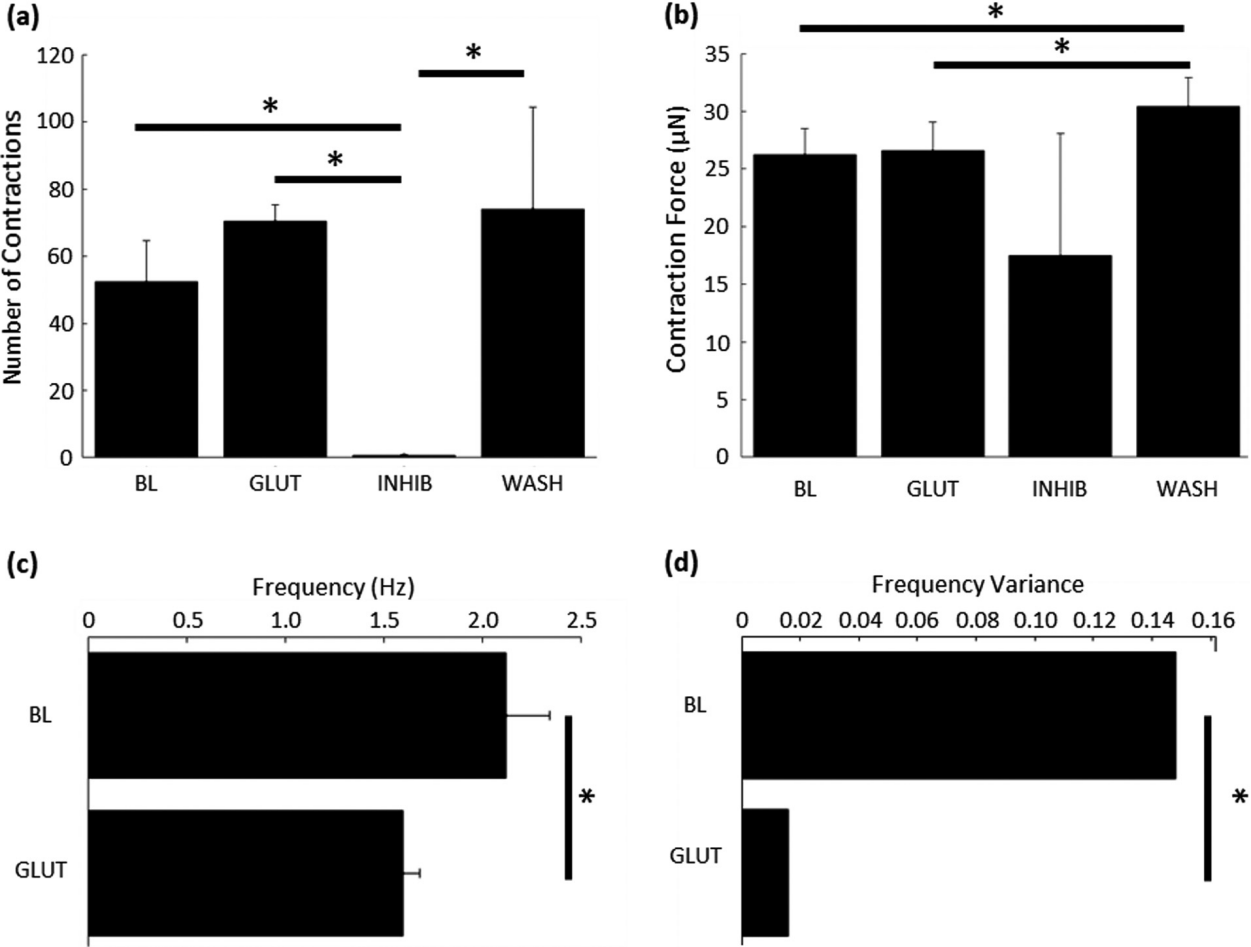
Glutamate signaling within the spinal cord mediates patterned muscle contraction. (a) The overall number of muscle contractions did not increase when glutamate was added; however, inhibition of glutamatergic signaling reduced the number of contractions to nearly zero. Contractile behavior was recovered by washing out the inhibitors. (b) Glutamate application did not significantly change the force of muscle contraction from the baseline. Due to the very low number of contractions under inhibition, the force of those contractions is not significantly reduced. The contraction force fully recovered upon washing out the inhibitors. One-way ANOVA, Dunnett's test. (c) Spontaneous neuronal firing in the spinal cord at the baseline caused the muscle to contract at 2 Hz, and the addition of glutamate reduced the contractile frequency to 1.5 Hz. Mann–Whitney test, ^*^p < 0.05 (n = 3). (d) Glutamate-initiated contraction exhibited significantly less variable frequencies of contraction, indicating a consistent, patterned output from the spinal cord. Hartley's Fmax test, (n = 3 for all conditions). ^*^p < 0.05. Graphs (a)–(c) are mean + SEM.

Neuromodulatory effects of glutamate and glutamate antagonists on neuron-muscle co-cultures. To analyze the videos of muscle contraction, a region of interest (ROI) around one of the hydrogel pillars was isolated [Fig. 4(a)] and its displacement was plotted over time [Figs. 4(b)–4(d)]. The overall number of contractions in a given time-period was not significantly different between the baseline (BL; 52 ± 12) and glutamate (GLUT; 70 ± 4) conditions [Fig. 5(a)]. However, the DNQX/APV application resulted in 78-fold and 105-fold reductions in the number of contractions at the baseline and with glutamate, respectively. Upon washout, another post-washout baseline was recorded. Significantly more contractions were observed (WASH; 74 ± 30) than at post-inhibition (INHIB; 1.3 ± 1.8) but without any significant change from the baseline [Fig. 5(a)]. Pillar displacements from within the ROI were translated into force using Hooke's law. Contraction force was also not different between baseline (26 ± 2 *μ*N) and glutamate (26 ± 2 *μ*N) conditions [Fig. 5(b)]. The post-washout contraction magnitudes were slightly larger (30 ± 2 *μ*N) than those that occurred during the original baseline and glutamate conditions [Fig. 5(b)].

Although the application of glutamate did not result in a change in the number or magnitude of contractions, it did significantly alter the frequency and the variance of that frequency. The contractile frequency was measured as the inverse of the peak-to-peak inter-contraction interval. The frequency of contraction decreased by 20%, from 2.11 Hz at the baseline to 1.59 Hz with glutamate application [Fig. 5(a)]. Both these behaviors are within the known range of contractile frequencies observed *in vivo* (0.5–2 Hz).^46^ This decrease in frequency occurred without a concomitant increase in contractile magnitude [Fig. 5(b)] or a change in the number of contractions [Fig. 5(a)]. The primary driver of this difference in contractile frequency was a change in the patterning of neural stimulation. The variance of glutamate-stimulated contractions was decreased to only 10.67% of the baseline variance, indicating that spinal cord neurons produced a rhythmic firing pattern that generated the patterned muscle contractions. Electrophysiological recordings confirm that cultured spinal cord segments can produce rhythmic firing patterns as late as 16 DIV [Fig. S1(c)]. This rhythmicity indicates that there is a glutamate-sensitive pattern generating circuit within the spinal cord segment.

## DISCUSSION

Our study demonstrates the engineering of a first fully functional 3D neuromuscular junction using a physiologically intact spinal cord to drive patterned muscle contraction. The co-culturing of an intact spinal cord with a 3D muscle tissue resulted in a skeletomuscular system under neuronal control that not only responds to glutamate signaling but is also capable of undergoing patterned muscle contractions, a key component for any locomotor task. The sustained, patterned muscle contractions observed in response to tonic glutamate stimulation of the spinal cord suggest that the L1–L2 segment forms functional NMJs with the muscle and that the internal spinal cord circuitry of the CPG remains electrically active throughout the co-culture. Thus, our spinobot appears to mimic the partial development of the peripheral nervous system (PNS). This informs a future design space involving the coordination of multiple muscles into flexor-extensor pairings, tuning capabilities, or the ability to incorporate the sensory dorsal root ganglion as inputs for the spinobot.

Under conditions of culture, spinal cords project a complex arbor of motor neurons and glia that co-localize with nicotinic AChRs when paired with engineered muscle tissue. The neurotransmitter glutamate mimics excitation from upper motor neurons of the brainstem, which control and coordinate lower motor neuron firing. The engineered spinal cord-skeletal muscle construct contracts in response to glutamatergic neuronal stimulation. This behavior is nearly completely suppressed by the addition of glutamate receptor antagonists DNQX and APV. This confirms that the observed baseline contractions were driven by spontaneous spinal cord activity and that the spinobot itself exhibits a low level of spontaneous muscle twitching. The low spontaneous muscle twitching under glutamatergic inhibition is another hallmark of muscle innervation. Non-innervated muscle monocultures will twitch frequently and spontaneously, while innervated muscle demonstrates very few muscle-initiated spontaneous contractions.^47^ Finally, we show that muscle contractions exhibit more rhythmic patterning when spinal neurons are chemically excited than those at the baseline.

During normal embryonic development, motor neurons reach the muscle just as the myoblasts are fusing into multinucleated muscle cells and early myotubes, a timeline we approximated experimentally. In our experiments, the spinal cord was introduced to the engineered muscle tissue 10 days post-seeding (7 days after the muscle was switched to differentiation medium), after the muscle had finished compacting and the muscle fibers had begun to fuse. The developing NMJ forms as a result of bi-directional signaling between motor neurons and skeletal muscle fibers.^41,48^ Within minutes of initial contact, presynaptic motor neurons begin firing and release ACh into the synaptic cleft.^45,49,50^ This causes the postsynaptic muscle to begin producing retrograde signaling molecules, e.g., neuregulin, as well as synthesizing AChRs and reorganizing their distribution.^42,50–53^ Over about a week, synaptic vesicle production increases, cytoskeletal reorganization occurs on both sides of the synapse, and AChR clustering begins, along with many other changes.^52,54–56^ Our results demonstrate that the structural development of motor end plates in our 3D neuromuscular system closely matches the *in vivo* developmental program, which we hypothesize would not be possible without both retro- and anterograde signaling.^57^

A C2C12-derived skeletal muscle bioactuator approximates mammalian muscle contraction by mimicking the articulation of bones across flexible joints.^9^ To induce a locomotion-driving contraction of the muscle strip, the biobot was positioned inside of an electric field and subjected to pulsed stimulations, which resulted in large-scale contractions. Pulsed 1 Hz stimulation generated contractions with forces between ∼75 and 200 *μ*N. A second iteration of this biobot used C2C12 cells that were genetically modified to express a mutated variant of the blue light-sensitive ion channel Channelrhodopsin-2 (ChR2) (Raman *et al.*, 2016).^31^ These optogenetic muscle bioactuators produced significantly smaller contractile forces, between 25 and 45 *μ*N in response to 1 Hz stimulation. The authors posit that one reason for the reduced force may be that the penetration depth of the 470-nm light was only ∼600 *μ*m, about half of the maximum thickness of the muscle strip (∼1200 *μ*m). This rendered the optical stimulus unable to excite the entire muscle strip simultaneously. More recently, a novel figure eight-style design of a bioactuator powered by a C2C12-derived muscle was able to generate forces up to 1.1 mN via electric-field stimulation.^13^

*In situ*, innervated skeletal muscle contractions produce more force than the spontaneous contractions of an isolated muscle.^54^ However, early attempts at innervating C2C12-derived muscle strips with stem cell-derived motor neurons have not achieved this force. Previous research utilized mouse embryonic stem cell (mESC)-derived embryoid bodies (EBs) that undergo a motor neuron differentiation program to innervate 3D C2C12-derived muscle tissues. One method is to implant the EBs directly onto the muscle strip as it compacts, permitting them to co-differentiate. When stimulated with 200 *μ*M or 400 *μ*M glutamate, which excites the motor neurons, small muscle contractions of ∼6 *μ*N and ∼10 *μ*N were observed, respectively.^19^ Muscle contractions have also been measured in a microfluidic device that enables 3D co-culture of C2C12-derived muscle and optogenetic mESC-derived embryoid bodies.^23^ When mESC-derived embryoid bodies were optically stimulated, the downstream muscle exhibited maximal contractile forces of ∼0.5 *μ*N. By co-culturing motor neurons derived from human induced pluripotent stem cells with C2C12-derived myoblasts, stimulating the neurons with 0.1 mM glutamate generated contractile forces in the differentiated muscle tissue of ∼1 *μ*N.^30^ Here, we demonstrate spinal cord-driven contractions with a maximum force of ∼41 *μ*N, with the mean contractile force of ∼25 *μ*N. This notably larger force is likely due to the robust innervation of the muscle by the profusion of outgrowth from the spinal cord (Fig. 1).

All the derived muscle tissues contract less than native fetal muscle in rat, which can exhibit forces up to 16 N.^58^ Previous data have shown that C2C12-derived skeletal muscle contracts less than primary skeletal muscle *in vitro*.^44^ This likely accounts for some of the difference between engineered and native muscle. However, it has also been demonstrated that innervated skeletal muscle contracts with more force than muscle alone.^54^ We hypothesize that the large discrepancy between neuronally mediated contractions and direct stimulation of the muscle via electric fields or optogenetics is also due to the number of muscle fibers being recruited by the stimulus.^59^ In the neural stimulation paradigms, the innervation does not reach as many fibers as when the whole tissue is stimulated simultaneously by an electric field. However, we note that the spinal neurons produce stronger contractions than their stem cell-derived counterparts. This is likely due to increased motor neuronal innervation of the muscle tissue. Another explanation is that neuromuscular junction development occurs over approximately 3 weeks *in vivo,* while the platforms described here and elsewhere generally provide only 7–10 days of co-culture. As the NMJs approach full maturity, it is expected that the amount of spontaneous neural firing will decrease, more myotubes will become singly innervated, and stimulated muscle contractions will generate more force.^42,45,56^

A 3D spinal cord-skeletal muscle platform with functional and controllable neuromuscular junctions could serve a variety of uses across disciplines. It would be an excellent tool for studying the pathology of neuromuscular diseases and a platform for studying the efficacy and modes of action of novel drugs for treatment. We could also visualize this spinobot as a model for Amyotrophic Lateral Sclerosis (ALS), spinal muscular atrophy, Duchenne's muscular dystrophy, and peripheral neuropathies such as those secondary to diabetes mellitus. Some researchers have already begun adapting *in vitro* NMJ platforms for the study of these diseases.^30^ The modularity of the spinobot demonstrated here, innervation from a living spinal cord, and the use of an easily transfectable cell line are unique strengths of this platform. It is also possible that biohybrid robots and actuators will one day be used at the interface for human-computer devices or appear as surgical training tools.^60–62^ Other variations on 3D microphysiological systems have been developed with microfluidic chambers, neural stem cells, and/or photolithography control. However, these platforms all lack the ability to generate the patterned contractions we produce by stimulating the spinal cord, which houses the robust lumbar CPG. Notably, the contraction forces generated by spinal cord-controlled C2C12-derived muscle tissue were 25–40× greater than those for mESC control. The use of the spinal cord also enables a future design space involving the coordination of multiple muscles from a single neuronal source or the ability to incorporate the sensory dorsal root ganglion as inputs for the spinobot, allowing it to respond to environmental cues.

## METHODS

### Animal welfare

Animal procedures were developed in accordance with the Public Health Service Policy on Humane Care and Use of Laboratory Animals and reviewed and approved by the University of Illinois at Urbana-Champaign Institutional Animal Care and Use Committee (Protocol #18172). Cultures of primary hippocampal neurons were from postnatal day 1–2 Long-Evans BluGill rats from an inbred colony maintained by our laboratory, according to the previously established protocols.^63^

### Spinal cord extraction and seeding for 2.5D culture

A 35 mm Petri dish with a 14 mm glass coverslip bottom (MatTek Corporation, Ashland, MA) was coated for a minimum of 1 h in 0.1% gelatin (EMD Millipore, Burlington, MA). The gelatin was rinsed 3–5 times with sterile phosphate buffered saline (PBS), followed by the addition of 100 *μ*l of 1% Matrigel onto each glass coverslip. The Petri dishes were then placed in the incubator for 60–90 min to allow for Matrigel polymerization. For each dish, a single BluGill rat pup between postnatal days 1 and 5 (P1–P5) was decapitated. The vertebral column was rapidly dissected from the pup and cleaned to expose the vertebrae. A small section of the spinal column from the T13 to L2 vertebrae was isolated using a pair of small scissors. Then, making two diagonal cuts at ±45° from the ventral midline, the ventral bone was removed, thereby exposing the spinal cord. The spinal cord was then rapidly and gently dissected with a pair of fine-tipped tweezers.

The spinal cord was placed on ice in 5 ml HibernateA (Life Technologies, Gaithersburg, MD) where the dorsal root ganglia were removed. The spinal cord was moved to a laminar flow hood where it was rinsed with fresh HibernateA. Using forceps, the spinal cord was seeded with the ventral side facing down directly onto the polymerized Matrigel. The spinal cords were placed back in the incubator for 45 min to allow the spinal cord to settle before adding 5 ml of Spinal Cord Growth Medium (SCGM) consisting of 96.825% NeurobasalA (Life Technologies) without L-glutamine or phenol red (Life Technologies), 2% GS21 (MTI-GlobalStem, Gaithersburg, MD), 1% penicillin-streptomycin (Cellgro Mediatech, Inc., Herndon, VA), 0.125% Glutamax (Life Technologies), 0.025% Brain Derived Neurotrophic Factor (10 ng/ml, EMD Millipore), and 0.025% Nerve Growth Factor (1 ng/ml, EMD Millipore). All cells were maintained at 37 °C and 5% CO_2_, with half media replacement every 2–4 days. Half media changes were implemented to maintain the presence of secreted extracellular cues while still providing a fresh source of nutrients.

### Design and fabrication of parts

A commercial stereolithography apparatus (SLA, 250/50, 3D Systems) was modified for polymerization as previously described.^9^ Parts generated using computer-aided design software were exported to 3D Lightyear software (v1.4, 3D Systems), which sliced the part into layers. Prepolymer solutions for biobots and holders are described previously.^3^ For fabrication of biobots, an 18 × 18-mm^2^ cover glass was secured to the center of a 35-mm culture dish before fabrication. For biobot holders, cover glass slides were first treated with 2% (vol/vol) 3-(trimethoxysilyl)propyl methacrylate (EMD Millipore) in 200-proof ethanol (100% EtOH) for 5 min and then washed with 100% EtOH for 3 min, dried, and secured to the center of a 35-mm dish. Following fabrication, each structure was rinsed with PBS, sterilized in 70% EtOH for 1 h, and allowed to re-swell in PBS for at least 1 h. This protocol has been previously published with additional details.^9,64^

### Formation of muscle strip

C2C12 murine myoblasts were maintained in muscle growth medium (MGM) consisting of Dulbecco's Modified Eagle Medium with L-glutamine and sodium pyruvate (DMEM, Corning Cellgro), supplemented with 10% FBS (Lonza, Alpharetta, GA), 1% penicillin-streptomycin, and 1% L-glutamine (both Cellgro Mediatech, Inc., Herndon, VA) or muscle differentiation medium (MDM), which consisted of DMEM supplemented with 10% horse serum (HS, Lonza, Alpharetta, GA), 1% penicillin-streptomycin, and 1% L-glutamine. During cell seeding, C2C12 cells suspended in MGM were mixed with an ice-cold liquid solution of Matrigel (30% of total cell-matrix volume, BD Biosciences), fibrinogen (4 mg/ml, EMD Millipore), and thrombin from bovine plasma [0.5 U/(mg fibrinogen), EMD Millipore]. C2C12 cells were suspended in MGM at a concentration of 5 × 106 cells/ml and added to each holder in a total volume of 120 *μ*l unless otherwise specified. After 1 h, 4 ml of MGM was added. After 24 h, biobots were released from holders and switched to MDM with anti-fibrinolytic 6-ACA (EMD Millipore) and human IGF-1 (EMD Millipore) as noted. All cells and biobots were maintained at 37 °C and 5% CO_2_, with full media replacement every 1–2 days. This protocol has been previously published with additional details.^9,64^

### Spinal cord extraction and seeding on the biobot

For each biobot, a single neonatal spinal cord was extracted as described above. Biobots at 7 DIV were also brought into the hood at this time. The media were aspirated from the biobot Petri dishes and seeded with C2C12-derived muscle strips. The multicellular biobot was placed in a standard incubator at 37 °C and 5% CO_2_ for 90 min before adding 5 ml of co-culture medium (CCM) consisting of 50% NeurobasalA without L-glutamine or phenol red, 50% Dulbecco's Modified Eagle Medium with L-glutamine and sodium pyruvate, 10% FBS, 2% GS21, 1% penicillin-streptomycin, 0.125% Glutamax, 0.025% Brain Derived Neurotrophic Factor, and 0.025% Nerve Growth Factor. All cells and biobots were maintained at 37 °C and 5% CO_2_, with full media replacement every 1–2 days.

### Chemical stimulation and recording

The spinal cord was excised and placed in the recording chamber where it was submerged in 2 ml of CCM such that the dorsal side was just covered in media. Glutamate (stock, 10 mM) was prepared in 300 *μ*M aliquots in DMEM, frozen, and stored at −20 °C. APV and DNQX aliquots were also pre-prepared, frozen, and stored at −20 °C with stock concentrations of 50 *μ*M and 20 *μ*M in dH_2_0, respectively. The construct was allowed to rest in unsupplemented CCM for 5 min (Baseline). 20 *μ*l of glutamate was added to the CCM bath for 5 min (Stimulation). The CCM-glutamate mixture was washed out three times with pure CCM followed by CCM supplemented with 20 *μ*M APV and DNQX. After 1 min, 20 *μ*l of glutamate was added and recording continued for 5 min (Inhibition). At this point, three full media replacements were performed to wash out all chemicals from the chamber. The construct was once again recorded in unsupplemented CCM for 5 min (Washout). Finally, a 20 *μ*l bolus of glutamate was applied for 5 min. During each 5 min phase, a video camera was used to record all motions from time t = 0–60 s and t = 240–300 s.

### Immunofluorescence and histology

Tissues were rinsed with PBS and fixed in 4% (vol/vol) paraformaldehyde. Prior to immunostaining, tissues were permeabilized with 0.3% (vol/vol) Triton X-100 (EMD Millipore) and blocked with 3% Normal Goat Serum (NGS, Abcam, Cambridge, UK) for 30 min. Tissues were incubated with alpha-actinin (1:2000, Abcam), Beta-III Tubulin (1:2000, Abcam), conjugated alpha bungarotoxin (1:1000 EMD Millipore), or glial fibrillary acidic protein (1:20 000, Abcam) antibodies for 48 h at 4 °C and washed with PBS. Biobots were incubated with Alexa Fluor 488 goat anti-mouse IgG, Alexa Fluor 568 goat anti-chicken IgG, and Alexa Fluor 647 goat anti-rabbit IgG secondary antibodies (1:1000, Invitrogen, Waltham, MA) in PBS for 2 h at room temperature in the dark.

### Spinal cord electrophysiology

For each experiment, a single neonatal spinal cord was extracted as described above. The isolated spinal cord was submerged in physiological saline (composition in mM: NaCl, 129; KCl, 4; CaCl_2_, 2.5; MgCl_2_, 1.14; NaH_2_PO_4_, 0.5; NaHCO_3_, 25; glucose, 10, adjusted to pH = 7.4 with HCI) that had been superfused with oxygen (95% O_2_/5% CO_2_) before recording. The bath temperature was kept constant at 24 °C. Locomotor-like activity was induced by bath applying glutamate (10 mM stock, frozen, and stored at −20 °C). Neurons in the ventral horns were recorded with inspect-pin electrodes connected to a differential A/C amplifier (Model1700, A-M Systems, Sequim, WA) and a data acquisition system (PowerLab 8/30, ADInstruments, Dunedin, New Zealand). Records were digitized and recorded in LabChart 7.3 (ADInstruments) at a sampling rate of 20 kHz.

## STATISTICAL ANALYSIS

Statistical differences were determined by analysis of variance (ANOVA) followed by Tukey's post-hoc test for the magnitude and number of contractions, the Mann–Whitney U test for the frequency of contraction, and Hartley's Fmax for analyzing the variance of frequency, all with significance indicated by p < 0.05. The sample size is indicated within the corresponding figure legends. All data are presented as a mean ± standard error. Each study was repeated three times independently.

## AUTHOR'S CONTRIBUTION

C.D.K. and S.C.L. contributed equally to this work. C.D.K. designed and performed experiments, conducted analysis, and wrote this manuscript. S.C.L. designed and performed experiments. C.C. designed materials and performed SEM imaging and experiments. C.L. designed and performed experiments. G.N.K. performed experiments and ran statistical models. R.B. analyzed data. R.G. analyzed data. M.U.G. designed experiments and analyzed data. All the authors edited this manuscript. All the authors have given approval to the final version of this manuscript.

## SUPPLEMENTARY MATERIAL

See the supplementary material for spinal cord electrophysiology and movies of spinobot contraction.
